# Health for the Middle-Aged Artisan

**Published:** 1924-02

**Authors:** 


### HEALTH FOR THE MIDDLE-AGED ARTISAN.

### THE SOUL OF THE WORKER.

ON January 15, Dr. Major Greenwood, Medical Statistician to the Ministry of Health, lectured at Sheffield, in connexion with the winter's course on "Health Education," upon "The Preventable Mortality of Middle Age." The lecturer at the outset pointed to the significant fact that, although the English death-rate up to 45 is lower than that of Sweden, beyond that age it is much higher.

### Pneumonia as a Preventable Disease.

Continuing, Dr. Greenwood said: Let us accept Warrington and Bolton as fairly typical of industrial mortality conditions and take as our unit the whole registration county of Lancaster; and let us compare the rates of one of the great causes of mortality in later adult life, disease of the organs of respiration, other than consumption, of Lancashire and some other districts. In addition to bronchitis and pneumonia, heart disease and cancer have many victims in later middle life. Let us compare each of these three groups in a sample of industrial cities of the north and a sample of industrial cities in the midlands and south of England. I took out ten cities of the north—viz., Blackburn, Oldham, Preston, Bolton, Bradford, Burnley, Huddersfield, Leeds, Sheffield and Sunderland. In 1913 there were 168,076 men aged 45–55 in these cities, and of them 548 died of cancer, 840 of bronchitis and pneumonia, 601 of heart disease. Rates of 3.3, 5.0 and 3.6 per 1,000 living. I paired them off against 10 midland and southern business cities—Bristol, Coventry, Derby, Leicester, Norwich, Plymouth, Portsmouth, Southampton, Stoke-on-Trent, West Ham; working towns you observe, not watering-places or residences of well-to-do retired folk. These 10 towns had 144,076 men aged 45–65 in 1913; 422 died of cancer, 444 of bronchitis and pneumonia, 508 of heart disease; rates of 2.9, 3.1 and 3.5 per 1,000 living. The rates for heart disease are practically the same; for cancer the northern group has a little more than 10 per cent. excess; for pneumonia and bronchitis the excess is more than 60 per cent. Evidently pneumonia is to a large extent a preventable disease, and the North of England has still much to learn with regard to its prevention.

### East Ham Healthier than Oldham.

Let us think over those things a little carefully in attempting to answer that question—why do the older men die off so much faster in northern manufacturing industry than elsewhere? The county borough of Oldham and the county borough of East Ham had at the census of 1921 very similar populations—144,983 persons in Oldham, 143,246 in East Ham. One may perhaps say that Oldham is predominatingly a city of skilled operatives, East Ham of unskilled casually employed people. Probably people in Oldham have more money to spend; many more of its inhabitants can and do afford to spend a week at Blackpool of the inhabitants of East Ham who go to Margate or Brighton for their holidays; there is no annual holiday week in East Ham. But how did the adults of the two towns come off in the matter of dying in 1921? After early life is past, the East Ham death rate is enormously more favourable than that of Oldham, not a trivial difference, but a difference of fifty per cent. Is it possible that there is still some correlation between industrial efficiency and heavy mortality?

### The Emotional Life.

We find that there is an excessive mortality in later adult ages associated with the development of highly scientific industries. In a scientifically efficient industry it is every day more and more true that the workman is less and less a master of the whole craft, has less and less the joy of the creator or artist. Here, I believe, we reach the kernel of our problem. The men of 500 years ago, uneducated in our modern sense, intellectually undeveloped, often exposed to physical discomforts, to pain, hunger and thirst, which are a nightmare and a horror to read of, had an emotional life—a faith, as the theologians would say—an unconscious rejoicing in beauty and grace which we have wholly lost. We cannot bring back again this emotional life; at least we cannot bring it back in its old form. The emotions which found their satisfaction in the old forms of faith must be discharged through other channels. This fact has been recognised implicitly by all thoughtful men and women; it is the spiritual motive of the demand for improved facilities of education. Now the purpose of education, in the sense of my present argument, is not to equip men to carry out the technique of their jobs more efficiently, not even to enable them to earn more money; it is the far higher aim of enabling them to live better.

### The Education Which Saves Souls.

By the chances of life I am thrown among people, personal friends and intimates, who are mostly either my own age or some few years older, all well past forty. Some of these men have succeeded, as the world count success; others have failed. But they have all got something outside their daily round to which they can turn for pleasure. Their mere physical energies are failing; they have reached a time of life when the limitations of their possible worldly success are plain, but they have still saved up some incorruptible treasure. How many skilled artisans of 45 to 55 can taste of these pleasures? It is not that the immediate means are costly. The pursuit of algebra or the study of Plato—even since the rise of prices—is not expensive; a few visits to "the pictures" cost more than plenty of Greek books or pieces of paper to do sums on. What artisans have not got is the preliminary training enabling them to enjoy that pleasure. Many middle class men and women live an unhappy and thwarted life. But for many more of the artisan classes that life is obligatory. The education which saves souls is something very different from technical instruction; it is higher education in the former sense, which, as I believe, has a hygienic importance, and can prevent the loss of interest in life, the deterioration of mind and body which is one of the reasons why mortality in later adult life is high.

